# Piezoelectric Enhancement of Piezoceramic Nanoparticle-Doped PVDF/PCL Core-Sheath Fibers

**DOI:** 10.3390/nano13071243

**Published:** 2023-03-31

**Authors:** Zhangbin Feng, Ke Wang, Yukang Liu, Biao Han, Deng-Guang Yu

**Affiliations:** School of Materials and Chemistry, University of Shanghai for Science and Technology, Shanghai 200093, China; 202342993@st.usst.edu.cn (Z.F.); 213353169@st.usst.edu.cn (B.H.)

**Keywords:** coaxial electrospinning, core-sheath fibers, piezoelectric coefficient, piezoelectric nanoparticles

## Abstract

Electrospinning is considered to be an efficient method to prepare piezoelectric thin films because of its ability to transform the phase of the polymers. A core-sheath structure can endow fibers with more functions and properties. In this study, fibers with a core-sheath structure were prepared using polyvinylidene fluoride (PVDF) included with nanoparticles (NPs) as the shell layer and polycaprolactone (PCL) as the core layer. Their mechanical and piezoelectric properties were studied in detail. During the course of the electrospinning process, PVDF was demonstrated to increase the amount of its polar phase, with the help of nanoparticles acting as a nucleating agent to facilitate the change. PCL was chosen as a core material because of its good mechanical properties and its compatibility with PVDF. Transmission electron microscope (TEM) assessments revealed that the fibers have a core-sheath structure, and shell layers were loaded with nanoparticles. Mechanical testing showed that the core layer can significantly improve mechanical properties. The XRD patterns of the core-sheath structure fibers indicated the β phase domain the main component. Piezoelectric testing showed that the doped nanoparticles were able to enhance piezoelectric performances. The increases of mechanical and piezoelectric properties of core-sheath structure fibers provide a feasible application for wearable electronics, which require flexibility and good mechanical properties.

## 1. Introduction

Modern electronics are indispensable for human daily life, providing a variety of options for our civilization to develop into an intelligent world. For this reason, considerable attention has been paid to wearable electronics [1]. The rapid advancement of electrical technology leads to low consumption setup, for example, hand-worn gadgets such as smart watches [2,3]. However, the energy shortage and endurance become a big issue [4,5]. Usually, the batteries in wearable devices must to be lightweight and have high energy density and good durability [6,7]. Until now, most portable devices have been powered by the electrochemical energy stored in the battery; however, a battery that requires frequent charging could cause inconvenience, and at the end of battery life cycle, untimely recycling will lead to irreversible environmental pollution [8,9,10]. In an effort to solve this problem, researchers developed a self-charging battery that ingests energy from numerous sources, including solar energy [11], electromagnetic energy [12], and wind energy [13]. Batteries charged using such sources are typically unstable because they are affected by the environment, in contrast to energy harvesting through body movements, which has the advantage of being less affected by the environment [14]. Piezoelectric materials are deemed a potential solution as they are capable of collecting energy from the surrounding environment [15,16,17,18,19] such as vibration [20], body movement [14,21,22], and air flow [23].

Nanogenerators (NGs), flexible devices which could transform mechanical and thermal energy into electricity via the piezoelectric effect, can be used as sustainable and sufficient power, attributed to their light weight, flexibility, and ease of fabrication [24,25]. Lead zirconate titanate (PZT) is a first-generation piezoelectric material that is widely used [26] due to its high piezoelectric coefficient compared to other materials [27]. Yet, lead-based materials may be harmful to the environment [28,29]. Therefore, lead-free piezoelectric ceramics must be discovered. Piezoceramics mainly include zinc oxide (ZnO) [30], barium titanate (BaTiO_3_) [31], and strontium titanate (SrTiO_3_) [32,33]. However, the strong mechanical properties constrain the application for wearable electronic devices, even if they have a large piezoelectric effect. In contrast, due to their benefits of low density, flexibility, environmental friendliness, and strong biocompatibility, piezoelectric polymers, particularly PVDF and its copolymer trifluoro-ethylene (PVDF-TrFE), are regarded as the most probable options for flexible and wearable electronics [34].The crystallographic phases of PVDF are α phase, β phase, and γ phase. Most of the PVDF is made up of a nonelectroactive α phase. As a result, in order to convert the α phase into the electroactive β phase, stretching and high-electric-field poling are needed [35]. Although there are some other polymers exhibiting piezoelectric properties, including nylon, polylactic acid (PLA), polyurethane (PU), polyamides, and polyimide (PI), the piezoelectric performance of PVDF is far beyond them, which makes PVDF one of the most widely used piezoelectric polymers.

Electrospinning can be used for the creation of continuous fibers. It is one of the most effective and successful procedures for creating the inclusion of various polymers at the nanoscale level. With the exploration of electrospinning, the coaxial, triaxial and side-by-side electrospinning were developed in order to obtain different structure fibers, which are used in many fields [36,37,38]. The polymer solution moves toward the collector under the action of electric field and gravity [39], and in the process, it is stretched and whipped, and finally, fiber bundles are obtained. The process is the same for triaxial, solid needle electrospinning, and other types of electrospinning [40,41,42,43,44,45]. Ceramic nanoparticles contained in the solution can enhance the conductivity of the solution to a certain extent, which makes the spinning process easier. At the same time, nanoparticles can become nucleating agents of PVDF phase transition during electrospinning. According to a recent report from Yuan et al. [46], monoaxial particle-doped fibers from a single-fluid blending electrospinning exhibit a high piezoelectric coefficient and robust electromechanical coupling. The alignment of additional electrical dipoles and the generation of more β phase can be facilitated by nanoparticles contained in PVDF fibers [34].

Complex nanostructure and the related structure–performance relation represent one of the most important directions in nanoscience and engineering. The core-sheath and Janus structures are the most popular complex structures because they can combine two materials with different properties to realize the designed function [47,48,49]. Among all types of complex nanostructures, core-sheath is the most popular one [50,51,52,53], and core-sheath fibers have been widely investigated for their ability to isolate and load substances to enhance mechanical properties without side effects [54]. Coaxial electrospinning has been used to create functionalized core-sheath fibers by embedding or coating fibers with functional substances and the modification of surfaces and interfaces, leading to numerous applications in a wide variety of fields such as energy, environmental, drug delivery, tissue engineering, healthcare, and food engineering [55,56,57,58,59].

In this work, a new type of coaxial fiber was designed and fabricated to obtain a flexible piezoelectric film. PVDF included with piezoelectric nanoparticles and PCL act as sheath and core layers of fibers, respectively. The addition of nanoparticles enhances the piezoelectricity of the sheath. The core layer improves the mechanical properties of the fibers. The feasibility of coaxial composite fibers for wearable electronics was evaluated by the morphology, mechanical properties, structure, and piezoelectric properties test.

## 2. Materials and Methods

### 2.1. Materials

All the materials used in experiments were purchased: ZnO (Macklin, China, 99.9% metal basis, 30 ± 10 nm, M_W_ = 81.39), BaTiO_3_ (Macklin, China, 99.9% metal basis, M_W_ = 233.19), SrTiO_3_ (Macklin, China, 99.5% metal basis, <100 nm), PVDF pellets (Mw = 400,000 Da, Macklin, China), N, N-dimethylformamide (DMF, AR, ≥99.5%, China National Medicines Corporation Ltd., Beijing, China), acetone (AR, ≥99.5%, China National Medicines Corporation Ltd., Beijing, China), and PCL pellets (Merck, Mn = 80,000).

### 2.2. Fabrication of Core-Sheath Fibers Doped with Nanoparticles

**Figure 1:**
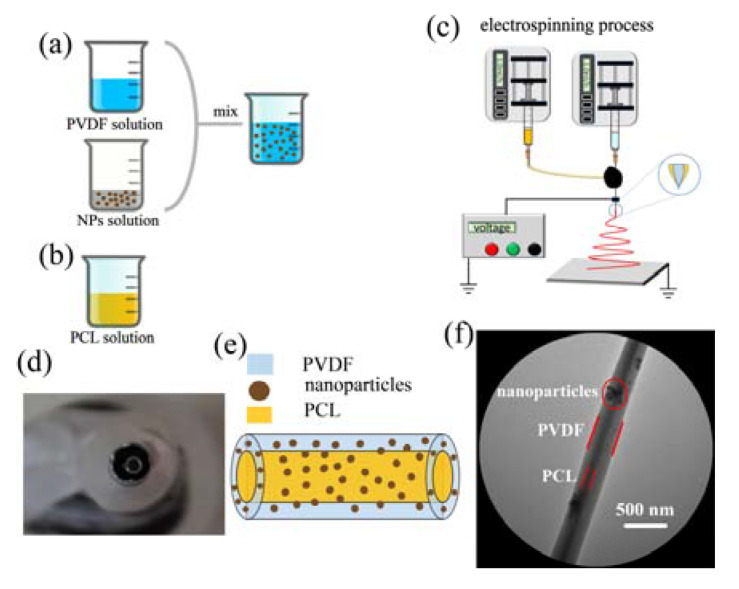
Schematic illustration of the electrospinning process. (**a**) NPs-PVDF solution. (**b**) PCL solution. (**c**) Electrospinning setup. (**d**) Homemade coaxial electrospinning needle. (**e**) Illustration of core-sheath structure fiber. (**f**) TEM image of core-sheath fibers.

In this paper, uniaxial electrospun PVDF fibers doped with nanoparticles are abbreviated as NPs-PVDF, and the core-sheath structure fibers doped with nanoparticles are labeled PCL/NPs-PVDF. The detailed diagram of NPs-PVDF solution fabricated process is presented in Figure 1a. In order to solve the agglomeration problem of nanoparticles in the PVDF solution, a uniform dispersed mixed solution was obtained. The nanoparticles and PDVF were weighed and dissolved in a DMF: acetone (3:2, *v*:*v*) solvent. The nanoparticles solution was magnetically stirred at room temperature, and the PVDF solution was pretreated by ultrasound. Then the nanoparticles solution was heated in a water bath at 60 °C and magnetically stirred for 5 h, then cooled to room temperature. The nanoparticles solution was added to the transparent PVDF solution and magnetically stirred until the mixture was uniform. The PCL was added to the DMF solution under magnetic stirring to obtain a 12 wt% solution, as shown in Figure 1b.

Figure 1c is a schematic diagram of a coaxial electrospinning device, including two pumps, two syringes, a high voltage power supply, a core-sheath needle, and an aluminum foil collector. The homemade core-sheath needle is shown in Figure 1d. The inner layer has an inner diameter of 0.84 mm, and the outer layer has an inner diameter of 2.85 mm. The inner solution of PCL and outer solution of NPs-PVDF were pumped separately. Aluminum foil connected to the negative electrode acted as a collector.

### 2.3. Characterization

X-ray diffraction (XRD) analysis of all materials was performed using an X-ray diffractometer (D8 ADVANCE, Bruker, Billerica, MA, USA), with a 5°/min scanning speed and a 2θ = 10–80° scanning range. TEM (Tecnai G2 F30 S-TWIN, FEI company, Hillsboro, OR, USA) was used to characterize the fiber structure. Infrared spectra were acquired using a Fourier infrared spectrometer (Perkin, CRUM 100). Scanning electron microscopy (SEM) (Bruker, Billerica, MA, USA) was used to observe the surface morphologies of the prepared fibers; the diameters of all samples were measured using ImageJ software. A universal material testing machine (Zwick/Roell Z020) was used to measure the mechanical properties. All the samples were cut to 20 mm width and 50 mm length for a standard shape at room temperature, and the test speed was 2 mm/min. To reduce error, every kind of fiber was tested three times, and the thickness was measured by a spiral micrometer. Testing the piezoelectric properties d33 was carried out by the PiezoMeter System (PiezoTest PM300, Singapore). The polarizing medium was dimethyl silicone oil, the poling temperature was 70 °C, and the film was polarized at 50 kV/cm for one hour. After poling, the film was naturally cooled to room temperature under the condition of constant poling voltage. Finally, the surface of the sample was cleaned with alcohol to remove the oil on the surface. Preparation of the gold electrode was performed by SEM gold-spraying equipment.

## 3. Results and Discussion

### 3.1. Implementation of Electrospinning Fibers

In this paper, two types of fiber membranes, uniaxial fiber membranes and core-sheath membranes, were electrospun. The uniaxial fiber membranes include pure PVDF fiber membranes and nanoparticle-doped fiber membranes. Coaxial fiber membranes use PCL as the core layer and PVDF with nanoparticles as the sheath layer.

**Figure 2:**
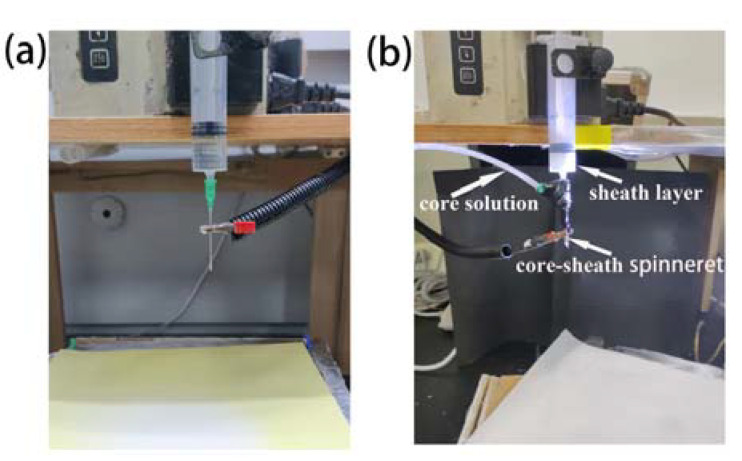
Electrospinning setup of (**a**) uniaxial electrospinning and (**b**) coaxial electrospinning.

In the process of uniaxial electrospinning, pure PVDF, ZnO-PVDF, BaTiO_3_-PVDF, and SrTiO_3_-PVDF solutions were used to fabricate the fibers. The electrospinning setup consisted of a pump, a syringe, a needle (18 G), a high-voltage supplier, and aluminum foil as the collector. After the solution was pumped from the syringe to the needle tip, a Taylor cone was formed under the surface tension of the solution and the electric field force. When the electric force exceeded the surface tension of the solution, the Taylor core was extruded into a straight jet. Then the positive charge on the straight jet split it into multiple fibers. Finally, the fibers were collected on the collector, as showed in Figure 2a. To form the nanoparticle-doped fibers, a homemade electrospinning system was assembled. The feeding rate and applied voltage were set about 2 mL/h 10 kV, respectively, and the distance between the needle tip and the collector was 15 cm.

The electrospinning process of coaxial electrospinning is a bit different from uniaxial electrospinning. The core solution and sheath solution are driven by the pump; then the two solutions meet at the end of homemade coaxial spinneret. Under the surface tension and electric field force, an inner and outer layer Taylor cone was formed. In the electric field force, the jet was extruded from the Taylor cone. Then core-sheath fibers were collected on the collector. The prepared core and sheath solutions were pumped separately. The core layer was NPs-PVDF, which functioned as the outer layer, and the PCL solution acted as the inner layer. The two solutions were pumped at a different speed from the electrospinning process. PCL was pushed at 1 mL/h, and the NPs-PVDF solution at 1.5 mL/h. The tip-to-collector distance was 15 cm, and the applied voltage was set at 12 kV. In the Figure 2b, coaxial electrospinning setups are captured.

### 3.2. Morphology of Electrospun Core-Sheath Fibers

**Figure 3:**
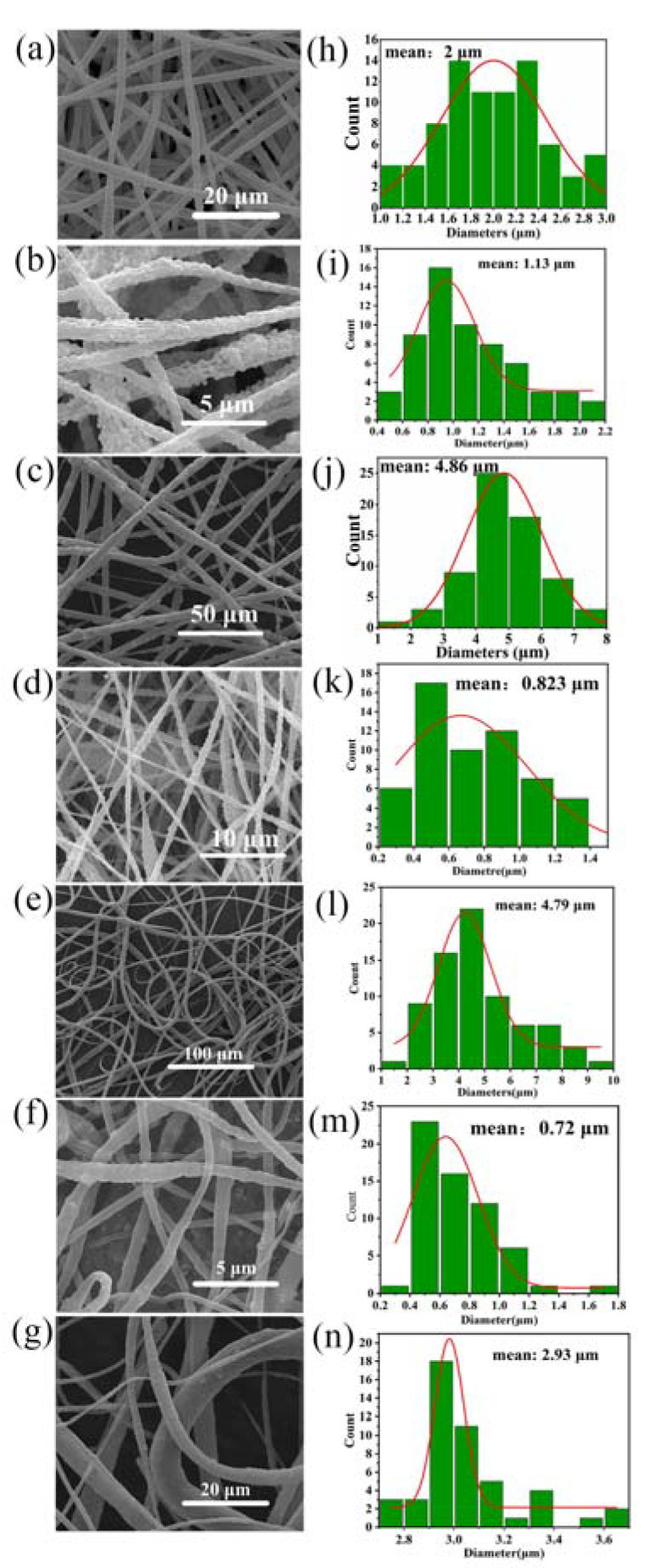
Fiber morphology by SEM: (**a**) pure PVDF fibers, (**b**) ZnO-PVDF, (**c**) PCL/ZnO-PVDF, (**d**) BaTiO_3_-PVDF, (**e**) PCL/BaTiO_3_-PVDF, (**f**) SrTiO_3_-PVDF, and (**g**) PCL/SrTiO_3_-PVDF. The distribution of electrospun fibers in (**h**) pure PVDF fibers, (**i**) ZnO-PVDF, (**j**) PCL/ZnO-PVDF, (**k**) BaTiO_3_-PVDF, (**l**) PCL/BaTiO_3_-PVDF, (**m**) SrTiO_3_-PVDF, and (**n**) PCL/SrTiO_3_-PVDF.

Figure 3 shows several SEM images of NPs-PVDF nanocomposites prepared by coaxial electrospinning with added piezoelectric nanoparticles. Figure 3a shows the SEM images of pure PVDF film prepared by electrospinning with a uniform fiber diameter of about 2 μm. Figure 3b,d,f show the PVDF with added nanoparticles. Interestingly, compared to pure PVDF fibers, the diameters of ZnO-PVDF, BaTiO_3_-PVDF, and SrTiO_3_-PVDF decrease, reaching 1.13 μm, 0.82 μm, and 0.72 μm, respectively. These show that the addition of nanoparticles is beneficial to obtain a fine diameter of fibers. This is because nanoparticles can enhance the electrical conductivity of the solution so that there is a greater electrostatic repulsion between fibers. This allows the fibers to be stretched sufficiently. It can be seen from the pictures that the uniformity of fiber thickness is greatly reduced, and the surface is rougher, which is due to the agglomeration of nanoparticles in the solution, which makes the continuity of the spinning process decrease.

The morphology of electrospinning fibers was influenced by many factors including solubility, charge density, electric field intensity, and surface tension of the solution. The addition of nanoparticles to PVDF can increase the solution conductivity, which, in turn, causes the electrical charge density at the surface of the droplet to rise during electrospinning as it forms at the tip of the spinneret. The electric field strength increases as a result, increasing the forces that stretch the droplet and encouraging the formation of finer fibers. Furthermore, the inclusion of nanoparticles can make the solution more viscous, which can encourage the growth of thinner fibers by delaying the start of the whipping instability [60].

However, the results were different for the coaxial fibers, as seen in Figure 3c,e,g. The diameters of PCL/ZnO-PVDF, PCL/BaTiO_3_-PVDF, and PCL/SrTiO_3_-PVDF core-sheath fibers rise sharply, reaching 4.86 μm, 4.79 μm, and 2.93 μm, respectively, nearly two times that of pure PVDF fibers and three to four times that of NPs-PVDF fibers. The increase in the diameter of coaxial nanofibers is the result of many factors The existence of the sheath layer surrounding the core material is one of the primary causes of the higher fiber diameter in the coaxial configuration. A polymer material with a higher molecular weight or viscosity than the core material can be used for the sheath layer. The sheath material can offer a stronger resistance to the electric field and stretching pressures during the electrospinning process, increasing the fiber’s diameter. Bead generation during the electrospinning process is another element that contributes to the increased diameter of the core-sheath fibers. Due to the higher viscosity of the sheath material, beads are more likely to develop in the coaxial structure, which increases the possibility of jet instability. The inclusion of beads may also cause the fiber’s diameter to rise.

### 3.3. Mechanical Properties

**Figure 4:**
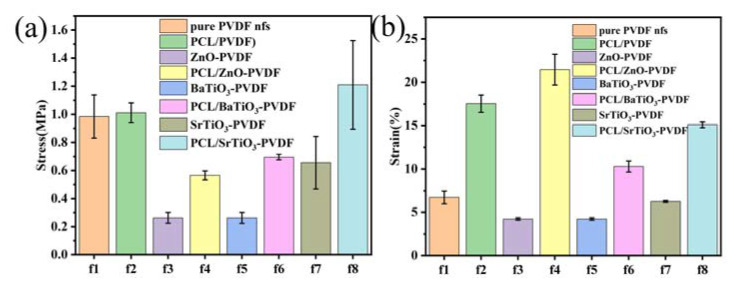
Mechanical properties of fibers. (**a**) Tensile strength of seven kinds of electrospun sheets. (**b**) Elongation failure of fibers.

The tensile strength of the individual fibers and their interfacial connection is crucial for the mechanical properties, which are typically evaluated as tensile strength and elongation failure strain. Tensile tests of PVDF fibers with added nanoparticles and fibers with a core-sheath structure were performed to determine how the additives’ effects on the mechanical properties of the fibers compared to control samples. Nanoparticle additives appear to worsen the mechanical properties of fibers. Figure 4 illustrates the tensile strength and elongation failure strain of PCL/NPs-PVDF fibers, pure PVDF fibers, and NPs-PVDF fibers.

Inclusion of nanoparticles is detrimental to the mechanical properties. By adding nanoparticles, the tensile strength decreased from 1.0 ± 0.1 MPa (pure PVDF fibers) to 0.26 ± 0.04 MPa (ZnO-PVDF), 0.26 ± 0.04 MPa (BaTiO_3_-PVDF), and 0.6 ± 0.2 MPa (SrTiO_3_-PVDF), which are declines of 73%, 73%, and 33%, respectively. The strain decreased from 6.7 ± 0.7% (pure PVDF fibers) to 4.2 ± 0.1% (ZnO-PVDF), 4.2 ± 0.1% (BaTiO_3_-PVDF), and 6.3 ± 0.1% (SrTiO_3_-PVDF). This result is consistent with that of Čech Barabaszová et al. [61]. The results proved that nanoparticles are harmful to the fibers’ strength by destroying the continuity. When nanoparticles are included in a polymer, the surface of the nanoparticles interacts with polymer molecules, which can lead to changes in the polymer molecule alignment. The cross-linking and physical links between polymer molecules may be broken, reducing the mechanical characteristics of the material, especially when the mass fraction of nanoparticles is significant. Moreover, the adding of nanoparticles may leave the material with flaws and pores that make it vulnerable to harm. As a result, the mechanical characteristics of electrospun PVDF may significantly decrease as a result of added nanoparticles [60].

Nevertheless, in core-sheath fibers, the tensile strength is 0.56 ± 0.03 MPa for PCL/ZnO-PVDF, 0.69 ± 0.02 MPa for PCL/BaTiO_3_-PVDF, and 1.2 ± 0.3 MPa for PCL/SrTiO_3_-PVDF. The strain is 21.4 ± 1.8%, 10.3 ± 0.6%, and 15.1 ± 0.3%, respectively. Core-sheath structure significantly improves the adverse effect of added nanoparticles. Regardless of the aspect of stress or strain, the mechanical properties of fibers with core-sheath structures have been significantly improved, which can be verified from Figure 4a,b. The addition of nanoparticles to PVDF may introduce defects and weaken the intermolecular interactions, leading to a reduction in the mechanical properties of electrospun fibers. However, when PCL is used as the core layer in coaxial electrospun fibers with PVDF as the sheath layer and nanoparticles added into PVDF, the mechanical properties of the fibers are significantly improved. This is because the PCL core layer can serve as a mechanical support for the PVDF sheath layer and prevent the deformation and collapse of the fibers, while the nanoparticles in the PVDF sheath layer can enhance the interfacial bonding between the two layers, resulting in a more uniform distribution of stress and strain. Moreover, the PCL core layer can also improve the flexibility and toughness of the fibers, making them less prone to breakage under external forces. In our work, the incorporation of PCL as the core layer in coaxial electrospun fibers with PVDF as the sheath layer can effectively improve the mechanical properties of the fibers [62].

### 3.4. Structure Characterization

**Figure 5:**
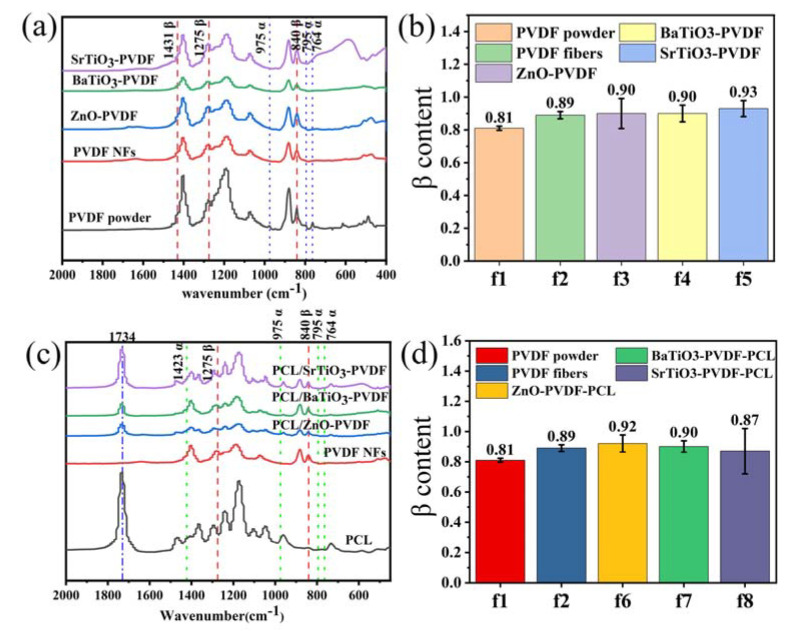
The FT-IR spectra and β content. (**a**) FT-IR spectra of PVDF with added piezoelectric nanoparticles. (**b**) β content of PVDF with added piezoelectric nanoparticles. (**c**) FT-IR spectra of core-sheath fibers with added piezoelectric nanoparticles. (**d**) β content of core-sheath fibers with added piezoelectric nanoparticles.

The formation of the polar phase in PVDF was confirmed by XRD and FT-IR spectroscopy analyses. Figure 5a represents the FT-IR spectra analysis of the PVDF powder, PVDF fibers, and ZnO-PVDF, BaTiO_3_-PVDF, and SrTiO_3_-PVDF fibers. For the PVDF materials, there are four characteristic absorption peaks of the nonpolar α phase at 764, 795, 855, and 975 cm^−1^ and three of the polar β phase at 840, 1276, and 1431 cm^−1^ [46,63]. All the α and β phases could be perfectly matched on the FT-IR. For the PVDF powder, we observed peaks at 764, 795, and 975 cm^−1^, corresponding to the α phase. For the electrospinning fibers, the observance peak of the α phase disappeared. These results confirm that electrospinning technology can significantly transform α phase into β phase, which is the key content for piezoelectricity [64]. Usually, the β content (F(β)) in the fibers is evaluated by the Lambert–Beer law [63],

$$F\left(\beta \right)=\frac{A_{\beta }}{\frac{K_{\beta }}{K_{\alpha }}A_{\alpha }+A_{\beta }}\times 100\%.$$

In this formula, A_α_ and A_β_ are the absorbance, corresponding to the α phase at 763 cm^−1^ and β phase at 840 cm^−1^, while K_α_ and K_β_ are the absorption coefficients corresponding to 6.1 × 10^4^ and 7.7 × 10^4^ cm^2^ mol^−1^. Figure 5b represents the β content of NPs-PVDF calculated by F(β).

The results show that the β phase content of electrospun fibers is significantly higher than that of raw materials, which indicates that electrospinning can promote the transformation from α phase to β phase. F(β) of PVDF powder is 81.2%, while the PVDF fibers reached 89.2%. This is attributed to the stretching and polarization during the electrospinning by transformation from the α phase to the alignment of the molecular dipole moment in the polymer chain. Moreover, ZnO, BaTiO_3_, and SrTiO_3_ nanoparticles added to PVDF fibers reach approximately 90.5%, 90.2%, and 93.4%, respectively. Figure 5d depicts the F(β) of PCL/NPs-PVDF. It is surprising that PCL/ZnO-PVDF, PCL/BaTiO_3_-PVDF, and PCL/SrTiO_3_-PVDF core-sheath fibers can reach 91.6%, 90.2%, and 87.6%, respectively.

**Figure 6:**
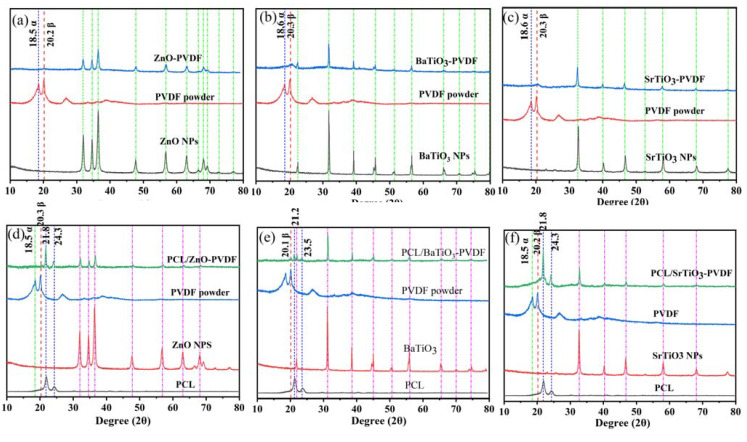
XRD spectra of (**a**) ZnO-PVDF, (**b**) BaTiO_3_-PVDF, (**c**) SrTiO_3_-PVDF, (**d**) PCL/ZnO-PVDF, (**e**) PCL/BaTiO_3_-PVDF, and (**f**) PCL/SrTiO_3_-PVDF.

The mainly β phase intense peaks of pure PVDF powder are at 20.3° and 36.3°, corresponding to the reflections of 110 and 020. The α phase with an intense peak is at 18.5°, which is illustrated in Figure 6. However, the presence of wide asymmetric peaks makes quantitative analysis of β content in fibers inexecutable since the peak is not obvious compared with nanoparticles. For the diffraction patterns of ZnO-PVDF fibers in Figure 6a, the peak at 18.5° corresponding to nonpolar α phase is flattened, while the β phase can be distinguished at 20.3°. This outcome is in agreement with FT-IR analysis. Moreover, there are additional diffraction peaks at 2θ = 31.9° (100), 34.6° (002), 36.4° (101), 47.1° (102), 56.8° (110), 63.0° (103), 66.5° (220), 68° (112), 69.2° (201), 72.6° (004), and 77.0° (202), which are associated with ZnO [65]. For the other two fibers with added BaTiO_3_ and SrTiO_3_, the diffraction peak of β phase can be clearly recognized from the XRD spectrum (Figure 6b,c), and the corresponding characteristic peaks of the NPs are also showed explicitly.

In Figure 6d, the black curve shows the XRD spectrum of PCL as having characteristic peaks at 2θ = 21.8° and 24.3°, respectively. The green curve is the XRD spectrum of PCL/(ZnO-PVDF) fibers, which exhibit PCL characteristic peaks. It proves that the PCL is successfully electrospun in the as-prepared films. The XRD spectrum of core-sheath PCL/ZnO-PVDF fibers shows slightly the peak of 20.3°, while there is no sign of α phase at 18.3°. The ZnO characteristic peaks can also be observed, proving that nanoparticles are electrospun in fibers. The same situation is observed in PCL/BaTiO_3_-PVDF and PCL/SrTiO_3_-PVDF core-sheath fibers (Figure 6e,f).

### 3.5. Piezoelectric Properties of Nanofibrous Films

By examining the existence of the β phase and the changes in piezoelectricity caused by the core-sheath structure, the origin of the improved electromechanical coupling for the core-sheath structure was investigated. To study the effect of nanoparticles in fibers on piezoelectric properties, a d_33_ piezoelectric coefficient meter was used. The results illustrated that the piezoelectric properties of PVDF fiber membranes have been significantly improved due to the addition of nanoparticles.

**Figure 7:**
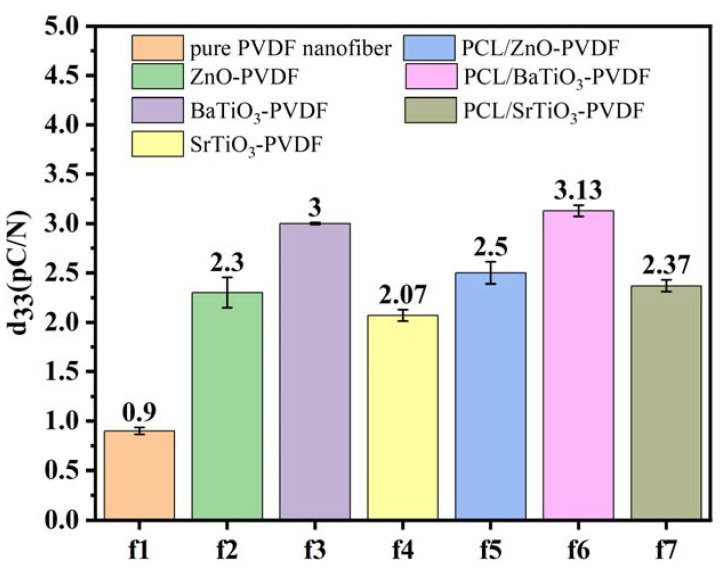
The d_33_ piezoelectric coefficient of electrospinning fibers.

As shown in Figure 7, the piezoelectric coefficient of pure PVDF fibers is 0.9 pC/N. ZnO-PVDF, BaTiO_3_-PVDF, and SrTiO_3_-PVDF fibers reached 2.3, 3, and 2.07 pC/N, respectively, which were enhanced by 159%, 233%, and 130%, respectively, compared with pure PVDF nanofibrous film. This indicates that the nanoparticles added to PVDF can significantly improve piezoelectric properties. As a ceramic material, piezoceramic acts as a nucleating agent in the formation of β phase in PVDF during electrospinning. This is because the presence of nanoparticles provides more surface area for the PVDF chains to adhere to, which can also function as physical barriers to stop the growth of unwanted α phase crystals. 

Moreover, the addition of PCL functioning as a core structure had no significant impact on the piezoelectric efficiency. The d33 piezoelectric coefficient of PCL/ZnO-PVDF fibers is 2.5 pC/N, that of PCL/BaTiO_3_-PVDF is 3.13 pC/N, and that of PCL/SrTiO_3_-PVDF is 2.37 pC/N. As a polymer, the piezoelectric effect of PCL itself is not strong, but when PCL acts as a core layer material inside the sheath layer of the piezoelectric material PVDF, the piezoelectric constant of the fibers increases due to the combination of the PVDF and PCL piezoelectric effect. In the core-sheath structure fibers, there is an interaction between the core materials and the sheath material, which can affect the piezoelectric properties of the fibers [66]. Moreover, in the d_33_ piezoelectric properties test, when external stress acted on coaxial fibers, the core layer material PCL deformed and interacted with the sheath layer material PVDF, which made the piezoelectric constant of the whole fiber increase.

These results indicate that the electrospinning is an effective method for improving the piezoelectric effect of PVDF because the electric field applied could rearrange the dipoles and mechanical stretch during the electrospinning process, which could promote the transformation of α phase to polar β phase.

Adding nanoparticles is an effective method to improve the piezoelectric properties of PVDF, which provides a potential method to fabricate flexible fibers.

## 4. Conclusions

In this article, the effects of nanoparticles (ZnO, BaTiO_3_, SrTiO_3_) and core-sheath structure on the morphology, mechanical properties, crystalline phase content, and piezoelectric constants of PVDF electrospun fibers were studied. PVDF fiber of NPs-PVDF and a core-sheath structure PCL/NPs-PVDF fiber were prepared. The results show that the addition of nanoparticles can increase the β phase content and piezoelectric constant of the fiber, but the mechanical properties of the fiber will be greatly reduced. However, the core-sheath structure can significantly improve the mechanical property loss caused by nanoparticles and has no effect on the β phase and piezoelectric constants. This is because nanoparticles will lead to holes and defects in fibers, which will destroy the continuity of the fibers. However, nanoparticles can be used as a nucleating agent to increase the content of β phase in fibers. The core layer in the core-sheath structure fiber can be used as the fiber skeleton, which significantly improves the mechanical properties of the fiber.

## Data Availability

Not applicable.
